# Balance between hydration enthalpy and entropy is important for ice binding surfaces in Antifreeze Proteins

**DOI:** 10.1038/s41598-017-11982-8

**Published:** 2017-09-19

**Authors:** Michael Schauperl, Maren Podewitz, Teresa S. Ortner, Franz Waibl, Alexander Thoeny, Thomas Loerting, Klaus R. Liedl

**Affiliations:** 1 Institute of General, Inorganic and Theoretical Chemistry, Center for Molecular Biosciences Innsbruck (CMBI), University of Innsbruck, Innrain 80-82, 6020 Innsbruck, Austria; 2 Institute of Physical Chemistry, University of Innsbruck, Innrain 52c, 6020 Innsbruck, Austria

## Abstract

Antifreeze Proteins (AFPs) inhibit the growth of an ice crystal by binding to it. The detailed binding mechanism is, however, still not fully understood. We investigated three AFPs using Molecular Dynamics simulations in combination with Grid Inhomogeneous Solvation Theory, exploring their hydration thermodynamics. The observed enthalpic and entropic differences between the ice-binding sites and the inactive surface reveal key properties essential for proteins in order to bind ice: While entropic contributions are similar for all sites, the enthalpic gain for all ice-binding sites is lower than for the rest of the protein surface. In contrast to most of the recently published studies, our analyses show that enthalpic interactions are as important as an ice-like pre-ordering. Based on these observations, we propose a new, thermodynamically more refined mechanism of the ice recognition process showing that the appropriate balance between entropy and enthalpy facilitates ice-binding of proteins. Especially, high enthalpic interactions between the protein surface and water can hinder the ice-binding activity.

## Introduction

Antifreeze Proteins (AFPs) are a structurally diverse class of proteins helping a variety of organisms, *e.g*. fish^1–5^, insects^6–8^, plants^9,10^, and bacteria^11^ to survive at temperatures below 0 °C^12^. AFPs lower the freezing temperature and slightly increase the melting temperature of water^13^. Therefore, these proteins are called *thermal hysteresis proteins*. The cryo-protection ability of those proteins also makes them interesting for industrial use like cryopreservation^14,15^, ice cream production^16^, frozen food storage^17^, and deicing^18^. Their structural variety indicates different evolutionary origins, whereby the specific face of the protein binding to the ice crystal, the ice-binding site (IBS), is similar for most of these proteins. The IBS is usually a rather apolar surface^19^ formed typically by threonine residues and, to a smaller extent, by other apolar amino acids (AAs) like valine (VAL), glycine (GLY), and alanine (ALA).

In order to understand how AFPs are able to prevent freezing, a variety of experimental methods have been applied to this protein family. The techniques include mutation studies^20^, ice-etching studies^20^, as well as structure determination methods like X-ray crystallography^21,22^ and NMR^23^. These have resulted in the currently accepted theory, stating that AFPs act by binding to the growing ice crystal^19^. This is remarkable and unique, as the protein’s natural solvent as well as ligand is water itself: in the liquid state acting as solvent, in the frozen state as ligand. Consequently, the binding of AFPs to ice-nuclei is likely one of the most challenging recognition problems posed by nature^24^.

AFPs prevent freezing by adsorbing to the growing ice crystal and subsequently inhibiting further ice growth^25–27^. The second step of this process, the inhibition, can be explained quite well with the principle of the Gibbs-Thomson (Kelvin) effect and is very well described in the literature^12,28^. In essence, the attached proteins force the ice surface to grow in a highly curved surface, resulting in a freezing point depression. Unfortunately, the adsorption mechanism is still not understood in detail^28^.

Computer investigations based on experimentally obtained, three-dimensional structures have been performed for an atomistic view on this problem. The first computational studies focused on the shape of the IBS^29–31^. They highlighted that the distances between regularly spaced residues at the IBS, or “active” site of an AFP, are comparable with the distances found between oxygen atoms in ice crystals^29–31^. This was also confirmed by subsequent reports and is still believed to be one of the key properties of IBSs^32,33^. Experimental observations confirmed this behavior, as AFPs were found to preferably bind to one specific ice site matching these IBS criteria^34^.

**Figure 1:**
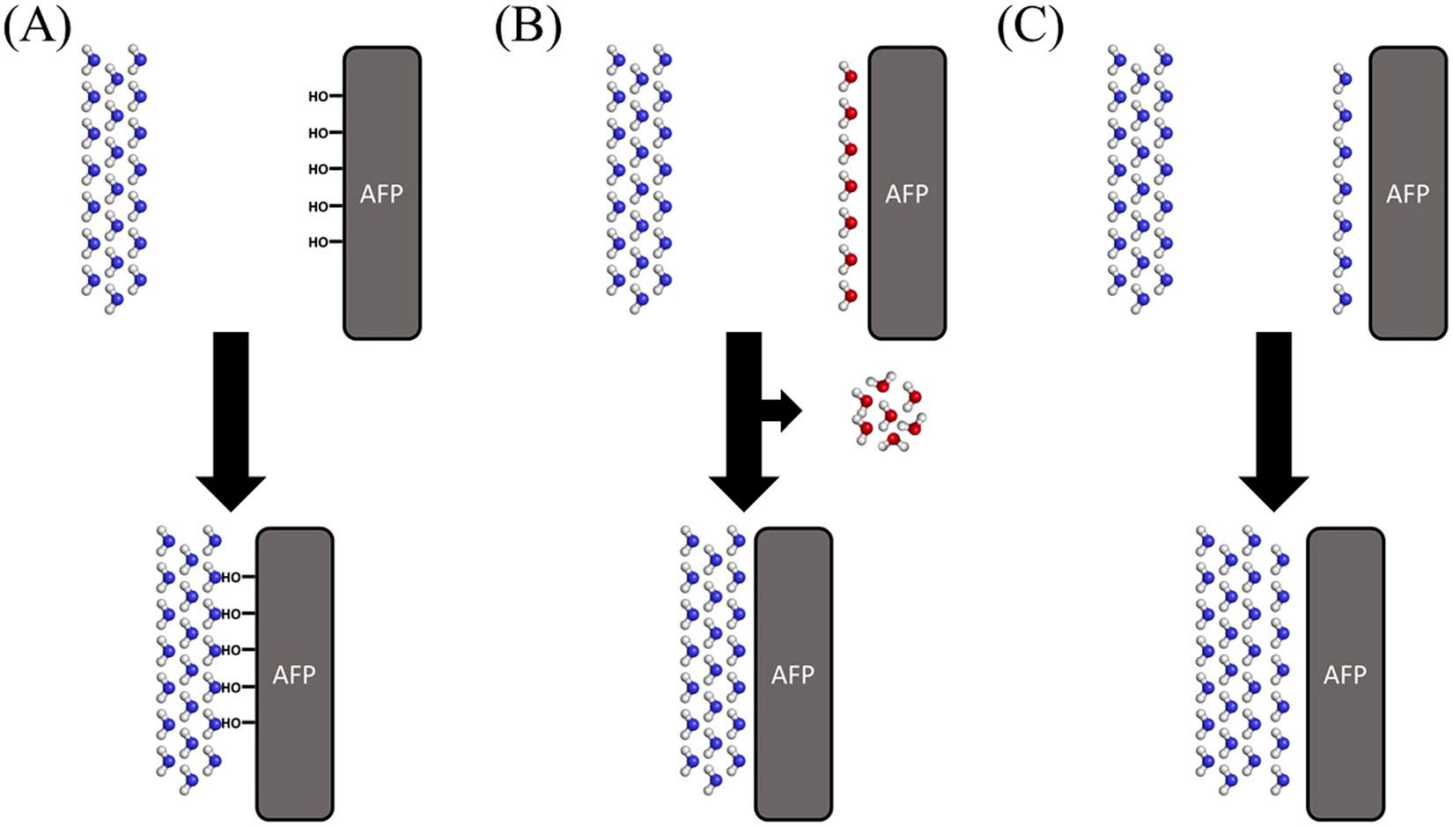
Possible mechanisms of ice-binding: (**A**) Hydrogen bonds are formed between the surface of the AFP and the ice crystal. (**B**) Entropically unfavored water molecules on the surface are removed upon the AFP binding to the ice crystal; the resulting entropy gain is the driving force. (**C**) The first hydration layer has an ice-shaped structure. The system strives to reduce the surface area of ice.

Nevertheless, not only the correct shape, but also the adequate interactions of the protein with ice and water play a significant role. Initial studies focused on the enthalpic part of the binding process and suggested that AFPs bind directly to the growing ice crystal *via* hydrogen bonds as sketched in Fig. 1A ^35,36^. The proposed mechanism was rejected soon, as experimental studies showed that the OH groups of the IBS only have a negligible role for ice-binding^4,5,37,38^.

Later on, several authors investigated the enthalpy gain upon binding of different AFPs to various crystallographic ice planes^28,39^. The enthalpy gain strongly depends on the positions of the hydrogen bond donors and acceptors. Still, for most IBSs the number of hydrogen bonds formed between the protein and liquid as well as between the protein and solid water is similar^40^. For this reason, a correct enthalpic fit of the protein surface might not be, as previously proposed, the only prerequisite to be fulfilled^41^. It was assumed that for AFPs the enthalpic interactions contributions for protein/ice and protein/water are similar^42,43^. This means no enthalpy is gained when the AFP binds to ice, and would render the concept of the enthalpy as the only driving force for ice recognition obsolete.

If enthalpy terms are similar, entropic contributions likely have to be the driving force for the ability of AFPs to detect and bind ice. The hydration of the AFP and the hydrophilic or hydrophobic character of its surface determine the entropic gain upon ice-binding. Furthermore, in real systems, AFPs have to diffuse through the ice-water-interface^44^. Therefore, simulations of AFPs already in contact with the ice surface might not reveal the full story of AFP binding. Sönnichsen *et al*. suggested that solvated AFPs contain entropically unfavorable water molecules bound to their surfaces. When the protein binds to ice, these water molecules are released into bulk, which results in an increase of entropy and therefore in a free energy gain (see Fig. 1B)^45^.

Conversely, Molecular Dynamics (MD) simulations showed that the residence time of water molecules around the IBS is too long for them to be removed upon ice-binding. Instead, they mediate the binding between ice and protein^33,46,47^. As a result, to date the most accepted theory states that AFPs are able to induce hydration shells resembling an ice surface (Fig. 1C)^33,42^. In order to minimize the surface between water and ice, which is favorable in terms of free energy, two ice surfaces bind to each other. However, for most substances in nature the hydration shell will not be a perfect ice surface, but rather an ice-like surface, showing deviations from the ideal ice surface geometry. This ice-binding mechanism was further refined by Kristiansen and Zachariassen^48^. They proposed that ice formation is favorable when water is trapped between two ice or ice-like surfaces. Thus, based on the theory that water molecules at an AFP surface are ordered as in ice, ice formation is favorable between the AFP “ice surface” and the growing ice crystal. As ice is the thermodynamically more stable phase, this phenomenon contributes favorable to the binding free energy^48^. In addition, this suggests that the ice is binding by growing towards the protein (ice-growth controlled), rather than the protein diffusing through the ice-water interface and then binding to the ice (diffusion controlled)^33^.

The proposed mechanism of ice-like water molecules was further investigated and refined by analyzing the dynamic behavior of water from simulations of AFPs with growing ice crystals^49^ and AFPs in liquid water^50,51^. Most of these more recent simulations focused on order and structure of the water molecules in the environment of this protein family^52^. It was shown that the pre-ordering of water in the first hydration layer is similar to structures found in various ice crystals^33,50^. Nevertheless, whether solely the first hydration layer is significant, or if ordering extends to higher layers, is still discussed controversially^53,54^. Typically, water molecules around AFP surfaces are less dynamic (higher ordering), compared to inactive surfaces^33,46^. Modig *et al*., however, found that the mobility of water molecules around AFPs is similar to proteins with no antifreezing activity^55^. The partial contradiction between these studies already suggests that the dynamics of the water molecules do not suffice to explain the mechanism of ice-binding for AFPs. Other properties like the hydrophobic or hydrophilic character of the surface might also be decisive^47^.

Although all these studies are partly contradictive, they sharpened our understanding of the general ice-binding mechanism. Nevertheless, a full, coherent explanation is still missing. Especially, the question whether or not the pre-ordering is the main and/or only driving force remains to be answered. Additionally, it is an open question why AFP surfaces do not consist of charged residues in a regular shape, as those AAs would increase the order of the water structure in comparison to the relatively apolar AAs often found in IBSs^56^.

This study combines the early idea of a significant enthalpy contribution with the more recent theory of the pre-ordering in the hydration shell, proclaiming an entropy gain upon binding to be the driving force for binding. We show that neither the pre-ordering nor the enthalpy alone can be used to explain the ice-binding ability of AFPs. Instead, we point towards the importance of balance between entropy, as indicator of the pre-ordering, and the enthalpy.

**Figure 2:**
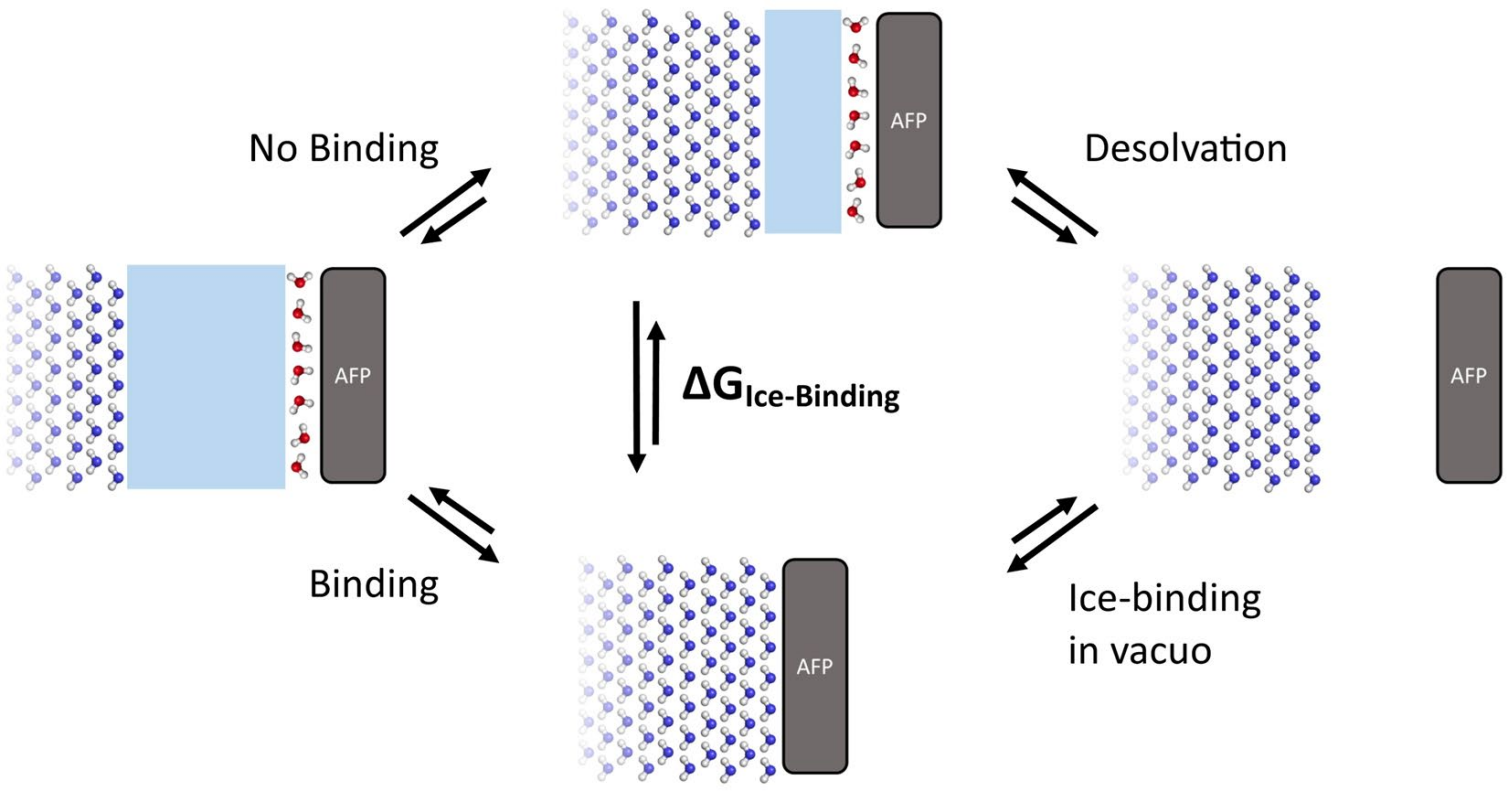
Schematic sketch of the ice-binding process: red water molecules represent the hydration shell of the protein, blue water molecules represent ice; light blue areas represent liquid bulk water. The energetic difference between ice growth in solution (top) and ice-binding to the protein (bottom) is highlighted by a thermodynamic cycle with two sub-processes: First, the desolvation of the ice-binding protein (investigated in this study); second, the binding of the ice-surface to the protein surface in vacuo.

To achieve this, we investigated the mechanism of AFP ice-binding from a more thermodynamic angle. Entropic and enthalpic contributions in the hydration shell of three different AFPs (from winter flounder (wfAFP), spruce budworm (sbwAFP), and mealworm (mwAFP)) were analyzed. By investigating three proteins of this structurally diverse family, we identified properties typical for the hydration shell of ice-binding proteins and their active sites. As known from drug design, the solvation and desolvation of binding sites can play an important role. Although the protein’s hydration shell is not removed, the hydration still changes from liquid water to ice. The energetic difference between ice growing towards an AFP (Fig. 2: bottom) and ice growing in water (Fig. 2: top) can be described by a thermodynamic cycle (Fig. 2: right): In the first step, the protein and the ice surface, binding to it, both lose their hydration shell. In the second step, the desolvated surfaces bind to each other. While the second step was intensively investigated in the literature, the first step, the desolvation, which is an essential part of the binding process, was very often neglected. In this contribution, we analyze and highlight which thermodynamic hydration properties facilitate ice-binding and which properties hinder the ice-binding activity. We show that although enthalpic interactions are similar in water and ice^43^, they can influence the ability of ice-binding significantly.

MD simulations of three AFPs in combination with Grid Inhomogeneous Solvation Theory (GIST) were used to study the hydration of AFPs. Inhomogeneous Solvation Theory has already been proven to be a valid approach to study the thermodynamics of water molecules surrounding proteins and peptides^57^. Furthermore, the grid-based approach of GIST allows investigating thermodynamic properties of water molecules with a spatial resolution. This permits evaluating the thermodynamics of single protein sites, helping to reveal why AFPs are able to detect ice in an excess of water.

## Results

To obtain an overview of the hydration thermodynamics around the three investigated AFPs, five representative, restrained conformations of every AFP were simulated and analyzed with GIST. To obtain free energy, entropy, and enthalpy values for the IBSs and the remaining protein surfaces, the contributions of all water molecules (grid points) in a rectangular layer with a thickness of 10 Å were summed up for three different AFPs (from winter flounder (wfAFP), spruce budworm (sbwAFP), and mealworm (mwAFP)), a cylindrical sector with a leg length of 10 Å was used. The acquired values describe the average thermodynamic contributions to the hydration pertaining to the respective protein surface. Mean values of the five conformations and the corresponding standard deviations for every protein site are given in Table 1. Definitions of the different protein surfaces, ice-binding site (IBS), rather ice-binding site (RIBS), rather non ice-binding site (RNIBS), and non ice-binding site (NIBS) are explained in the Methods section.

**Table 1:** Thermodynamic values of the water molecules around the AFPs at 260 K.

AFP	Site	Total Free Energy ΔG_Solv_	Total Entropy TΔS	Total Enthalpy ΔE	Solute-Water Enthalpy ΔE_SW_	TΔS/ΔE_SW_
wfAFP	*IBS*	*−2.8* ± *1.1*	*−32.9* ± *1.1*	*−35.7* ± *1.4*	*−64.3* ± *3.6*	*0.51* ± *0.02*
	*RIBS*	*−3.5* ± *1.2*	*−26.6* ± *0.5*	*−30.0* ± *1.0*	*−58.4* ± *2.7*	*0.46* ± *0.03*
	**RNIBS**	**−22.6 ± 3.8**	**−29.7 ± 1.4**	**−52.3 ± 5.1**	**−97.8 ± 10.4**	**0.31 ± 0.02**
	**NIBS**	**−50.2 ± 6.6**	**−40.7 ± 1.4**	**−90.9 ± 8.0**	**−166.1 ± 15.3**	**0.25 ± 0.01**
sbwAFP	*IBS*	*−6.7* ± *1.4*	*−51.2* ± *3.1*	*−57.9* ± *2.3*	*−118.3* ± *4.8*	*0.43* ± *−0.03*
	**NIBS1**	**−80.7 ± 2.0**	**−69.1 ± 4.3**	**−149.8 ± 5.6**	**−295.6 ± 11.4**	**0.23 ± −0.02**
	**NIBS2**	**−72.0 ± 2.9**	**−72.1 ± 2.7**	**−144.1 ± 5.4**	**−275.3 ± 9.3**	**0.26 ± −0.01**
mwAFP	*IBS*	*−5.6* ± *1.4*	*−48.1* ± *2.7*	*−53.7* ± *2.9*	*−101.7* ± *4.3*	*0.47* ± *−0.03*
	**NIBS1**	**−39.3 ± 3.3**	**−47.0 ± 1.4**	**−86.3 ± 3.6**	**−160.0 ± 7.4**	**0.29 ± −0.02**
	**NIBS2**	**−48.4 ± 2.9**	**−47.2 ± 1.0**	**−95.7 ± 3.6**	**−173.6 ± 6.9**	**0.27 ± −0.01**
	**NIBS3**	**−40.0 ± 3.5**	**−43.1 ± 2.2**	**−83.0 ± 3.9**	**−166.5 ± 8.7**	**0.26 ± −0.02**

All values are given in kcal/mol. Italic values describe the IBSs and bold values the NIBSs.

All IBSs show less negative values for the total free energy of solvation than the NIBSs (*cf*. Table 1 Column 3: italics compared to bold values). This agrees with chemical intuition, as IBSs are apolar, rather hydrophobic surfaces. The protein areas not directly binding to the ice (NIBSs) are often more hydrophilic, as they include charged and/or polar residues leading to a more favorable solvation free energy. Hence, these sites are responsible for the overall high solubility of the AFPs. The free energy of solvation calculated with GIST only covers contributions of water molecules as well as the direct interaction between the protein and the surrounding water. Intermolecular enthalpic and entropic differences of the protein resulting from solvation are not considered within the GIST analysis, but these contributions can only be calculated for the whole protein and would not be assignable to the individual protein sites.

The entropy of the water molecules can, in a first approximation, be correlated to their order within the hydration shell. As most of the more recent studies focused on the entropic part of solvation (pre-ordering of water molecules)^52^, it is rather surprising that no obvious trend for the IBSs in comparison to the other protein sites could be found (*cf*. Table 1 Column 4). In contradiction to other studies, which suggested that entropic terms, respectively high (ice-like) order could be important for the ice-binding surface^38^, our analysis shows an opposite trend for wfAFP and sbwAFP: The water molecules around the IBSs of the two proteins display less negative entropy values (lower order; *e.g*. −51.2 kcal/mol for swAFP) than those surrounding the NIBSs (−69.1 and −72.1 kcal/mol for sbwAFP). For mwAFP, the total entropy values are similar for the IBS (−48.1 kcal/mol) and the NIBSs (−43.1 to −47.2 kcal/mol). This already indicates that entropy alone is likely not a good indicator of ice-binding activity. IBSs consist of relatively apolar AAs (*e.g*. THR, ALA)^19^ in contrast to NIBSs. These AAs show higher hydration entropies (lower order) in free solution than charged and polar AAs^56^. This further supports our conclusion that besides the entropy other factors are of major importance for ice-binding activity, too, as otherwise IBSs should consist predominantly of the most apolar AAs *e.g*. PHE, TRP.

In contrast to the entropic contributions, Table 1 shows a significant trend in the enthalpic interactions of all IBSs compared to all other investigated protein surfaces (Table 1 column “Total Enthalpy”). The solvation enthalpy of the ice-binding surface is significantly less favorable than for the protein sites not interacting with ice. These low enthalpic interactions originate from relatively weak interactions between the IBS and its surrounding water (Table 1 Column “Solute Water Enthalpy”) as a consequence of the hydrophobic character of the involved binding sites.

The relation between entropy and enthalpy was further investigated. Strong enthalpic interactions are typically associated with high entropic restrictions within the phase space occupied by water molecules. Weak enthalpic interactions are associated with freely moving water molecules. This phenomenon is known as entropy-enthalpy compensation^58–60^. While for the IBSs entropy/enthalpy ratios between 0.43 and 0.53 (Table 1 last column) were obtained, for all NIBSs according values lower than 0.3 were found. This indicates that IBSs order the participating water molecules more strongly in comparison to other “non-functional” protein surfaces showing the same magnitude of enthalpic interactions. As a result, the ordering might still be one important piece of the puzzle – but it has to be investigated in combination with enthalpic contributions.

In contrast to other studies suggesting that the long-range order is significantly higher for IBSs than for other proteins^53^, our analysis shows an effect on the solvation entropy only up to 10 Å. The entropy terms are declining faster for the IBSs (bulk value is reached after approx. 9 Å) than for non active surfaces (bulk value is reached after approx. 11 Å).

**Figure 3:**
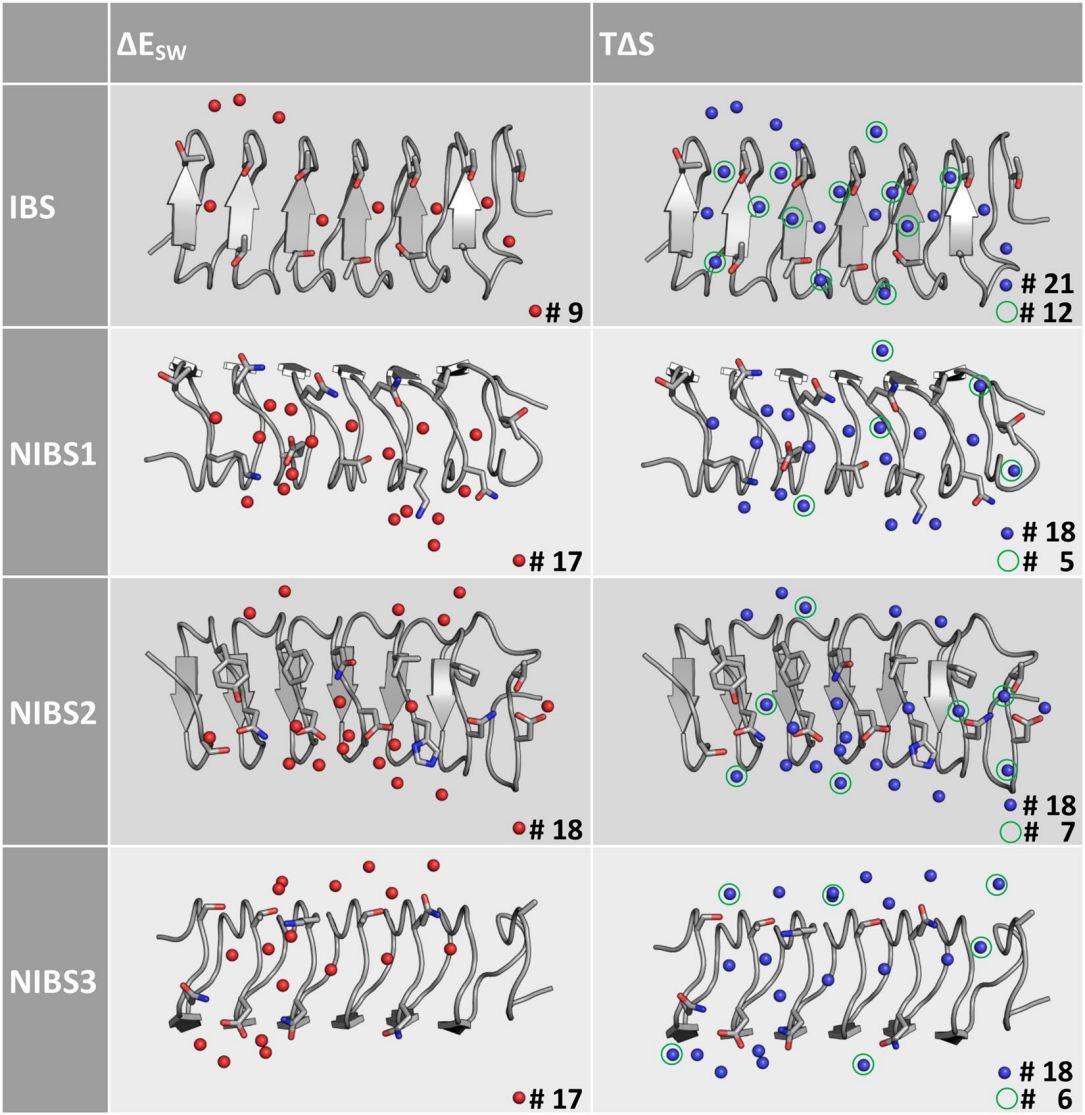
Water positions showing strong enthalpic interactions with the protein mwAFP are depicted as red spheres, those with unfavorable entropic values as blue spheres. The number of entropically unfavored water molecules is similar for all four sites; for the IBS there are fewer water molecules with favorable solute water enthalpy. Water molecules with unfavorable entropy and no enthalpic compensation are highlighted with green circles.

Further insight can be gained when instead of the overall entropic or enthalpic contributions of all water molecules close to one protein site, the thermodynamic properties of single water molecules are investigated. Therefore, all water molecules with an entropy term (TΔS) lower than −2.5 kcal/mol (Fig. 3 right) together with all water molecules of solute water enthalpy (ΔE_SW_) lower than −6.0 kcal/mol (Fig. 3 left) in the proximity of mwAFP were plotted. The water positions were derived from the grid water population and are therefore independent of the thermodynamic property (*e.g*. entropy or enthalpy) plotted. Hence, a water molecule shown in both plots corresponds to a water molecule with low (unfavorable) entropic interaction but also with strong enthalpic contributions. While the number of entropically unfavored (ordered) water molecules is similar for all protein surfaces, the number of strongly bound water molecules is significantly lower for the IBS (#9) compared to the remaining surfaces (#17–18). Furthermore, the IBS has more water molecules with unfavorable entropy values without enthalpic compensation (green circles, #12), whereas for the other surfaces fewer according water molecules can be found (#5–7). Similar trends are also observed for the other AFPs (sbwAFP and wfAFP). The orientations of the sidechains differ between the clusters, but have no significant influence on the obtained solvation thermodynamic results. However, a regular arrangement likely is an additional requirement for ice-binding activity^61^.

To further confirm the observed trends, experimentally tested mutations of the AFP of the winter flounder were investigated. The four THR (residue-id = 2, 13, 24, 35) were changed to four serine (SER) (Mutation SSSS), ALA (further called AAAA), or VAL (VVVV) residues. The SSSS modification shows no antifreezing activity at all, whereas the mutants AAAA and VVVV show slightly decreased antifreezing ability compared to the naturally occurring form (TTTT)^47^. In accordance with results obtained for the three different, original AFPs, similar trends were found for the mutations as shown in Table 2. The inactive mutation SSSS shows the strongest enthalpic interactions with the surrounding water for the IBS (total enthalpy of −40.4 kcal/mol; solute water enthalpy of −75.4 kcal/mol). Also, the entropy/enthalpy quotient is again lowest for the inactive species (0.39). The entropy shows no significant difference, except for the NIBSs displaying more negative values than all other sites. This can be attributed to the occurrence of charged AA sidechains in this region.

**Table 2:** Thermodynamic values of the wfAFP in its natural form (TTTT) and of the three mutations in kcal/mol at 260 K. Values for the IBSs are highlighted.

Mutation	Site	Total Free Energy ΔG_Solv_	Total Entropy TΔS	Total Enthalpy ΔE	Solute-Water Enthalpy ΔE_SW_	TΔS/ΔE_SW_
Wild-type	**IBS**	**−2.8 ± 1.1**	**−32.9 ± 1.1**	**−35.7 ± 1.4**	**−64.3 ± 3.6**	**0.51 ± 0.02**
(TTTT)	RIBS	−3.5 ± 1.2	−26.6 ± 0.5	−30.0 ± 1.0	−58.4 ± 2.7	0.46 ± 0.03
	RNIBS	−22.6 ± 3.8	−29.7 ± 1.4	−52.3 ± 5.1	−97.8 ± 10.4	0.31 ± 0.02
	NIBS	−50.2 ± 6.6	−40.7 ± 1.4	−90.9 ± 8.0	−166.1 ± 15.3	0.25 ± 0.01
SSSS	**IBS**	**−10.9 ± 0.6**	**−29.4 ± 1.3**	**−40.4 ± 0.9**	**−75.4 ± 2.6**	**0.39 ± 0.01**
(inactive)	RIBS	−7.3 ± 0.7	−25.3 ± 0.5	−32.7 ± 1.1	−62.2 ± 3.4	0.41 ± 0.02
	RNIBS	−18.7 ± 3.3	−26.7 ± 0.8	−45.4 ± 4.1	−85.7 ± 8.8	0.32 ± 0.02
	NIBS	−56.1 ± 6.6	−41.2 ± 0.5	−97.3 ± 6.3	−177.7 ± 12.4	0.24 ± 0.02
AAAA	**IBS**	**−5.1 ± 0.2**	**−29.3 ± 3.1**	**−34.3 ± 3.1**	**−60.8 ± 2.6**	**0.48 ± 0.01**
	RIBS	−7.2 ± 1.2	−24.2 ± 0.8	−31.5 ± 1.3	−59.4 ± 3.4	0.41 ± 0.02
	RNIBS	−16.7 ± 1.7	−28.0 ± 2.0	−44.7 ± 3.7	−83.6 ± 8.8	0.34 ± 0.01
	NIBS	−57.4 ± 5.9	−42.6 ± 1.2	−100.1 ± 5.6	−184.4 ± 12.4	0.24 ± 0.02
VVVV	**IBS**	**−2.8 ± 1.9**	**−28.0 ± 1.3**	**−30.8 ± 3.0**	**−57.1 ± 2.6**	**0.50 ± 0.03**
	RIBS	−6.8 ± 1.1	−25.3 ± 0.7	−32.0 ± 0.6	−61.6 ± 3.4	0.41 ± 0.02
	RNIBS	−18.7 ± 3.2	−30.1 ± 0.9	−48.8 ± 3.9	−90.8 ± 8.8	0.34 ± 0.02
	NIBS	−56.2 ± 6.3	−40.2 ± 0.7	−96.4 ± 6.7	−178.0 ± 12.4	0.23 ± 0.02

**Figure 4:**
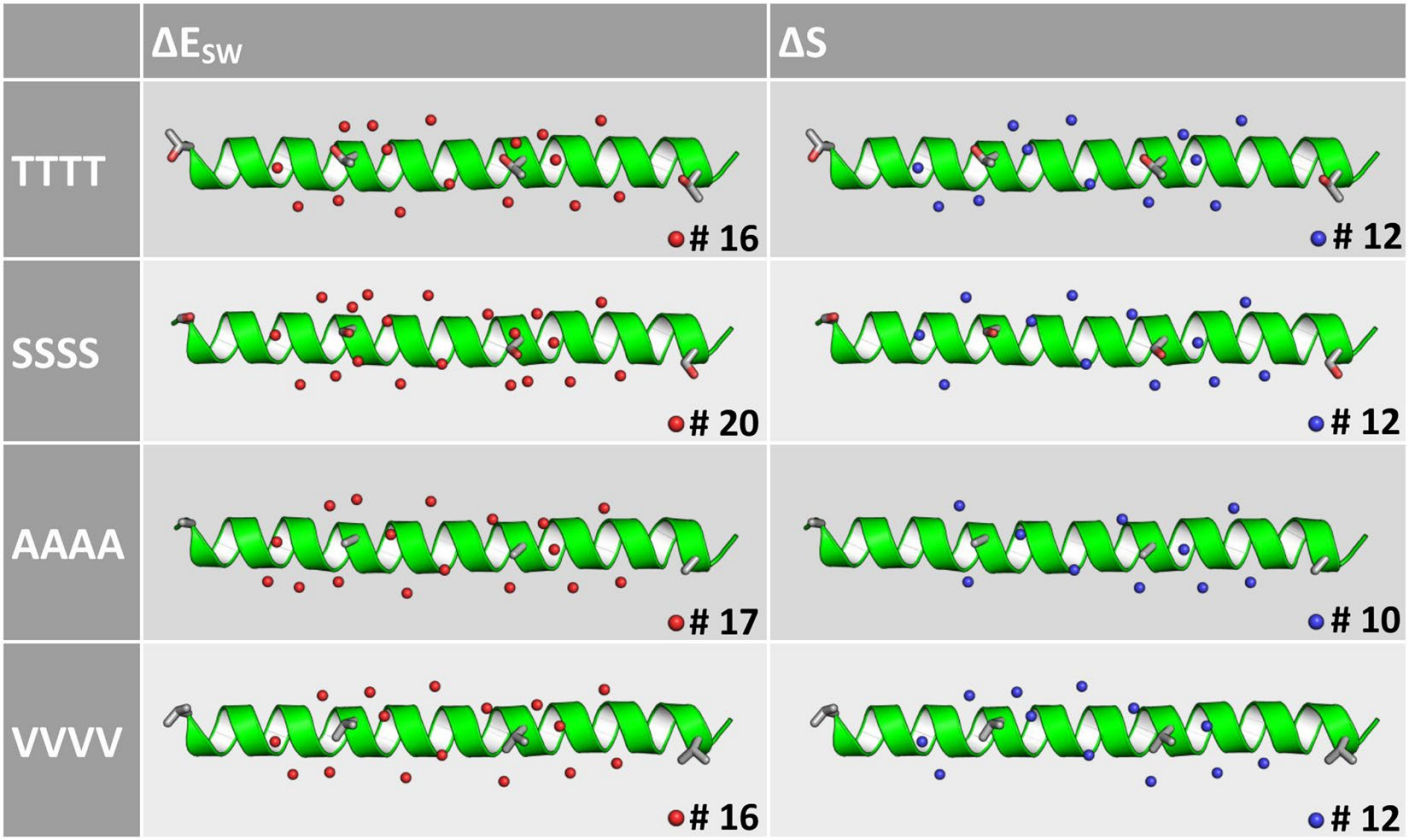
Water positions with strong enthalpic interactions are shown on the left (red spheres) and with unfavorable entropic values on the right side (blue spheres) for the naturally occurring form of wfAFP (TTTT) and three mutants. The four THR and the respective mutated residues are shown as grey sticks. The number of entropically unfavored water molecules is similar for all four sites, whereas for the inactive mutant SSSS the number of strongly interacting water molecules is increased.

Similar to the analyses for the three, naturally occurring AFPs, it is possible to examine the properties of single water molecules. Again, all enthalpically strongly interacting water molecules in close proximity to the two THR residues (or their mutation analogs) are shown as red spheres (Fig. 4 left), all ordered water molecules are shown as blue spheres (Fig. 4 right). In contrast to the original molecule, the solute water enthalpy (ΔE_SW_) cutoff was set to −3 kcal/mol/water molecule - as the protein is smaller and therefore allows for an inclusion of more water molecules. The entropic (TΔS) cutoff value is set to −2.5 kcal/mol/water molecule. Again, the number of strongly interacting water molecules is higher for SSSS (#20) than for all other forms (#16–17), whereas the number of entropically ordered water molecules is similar for all proteins (#10–13).

## Discussion

Many properties of a protein or its surface have to be within a specific range so that antifreezing activity, or more generally ice-binding, is possible. Firstly, the sidechains on the protein’s surface need to have a spacing similar to ice – a fact already well studied^29–31,62^. Secondly, the water molecules need to form an ice-like structure around the protein, which has been discussed extensively in studies on the pre-ordering and dynamics of water^33,50^. As all interactions between the protein and the rigid ice surface can also be formed between the protein and the flexible water molecules of the liquid phase, the binding enthalpy cannot be the driving force for the ice-binding process. Therefore, the entropic gain upon binding and/or the enhanced ice formation between the ice-like surface of the protein and the ice-surface are responsible for the ice-binding process to be energetically favorable. Surprisingly, our study could not find lower entropy values (higher order) in the IBSs compared to NIBSs. In agreement with studies by Modig *et al*., we find that IBSs do not show more pronounced long range hydration effects compared to other protein surfaces^55^. This strongly suggests that other forces also play a significant role. As the free energy, consisting of enthalpy and entropy terms, is the driving force for all chemical processes, it seems obvious that enthalpic interactions, although they are similar for protein-water and protein-ice, can have an impact on the ice-binding ability. Enthalpic interactions were more recently paid less attention to in the analyses of AFPs, for it was found that interactions between protein and ice are similar to those found in IBSs and water^40^. We, however, reveal that the enthalpy gains are significantly lower for IBSs than for other protein sites. On one hand, this is not surprising as the IBSs are more apolar than other protein surfaces. On the other hand, typically not the most apolar AAs are found in IBSs. The necessity for a perfect match between enthalpy and entropy in combination with the correct geometry is thus indicated. Our studies focus on the hydration properties of the AFPs and derive conclusions from this pre-ice-binding state (Fig. 2). We see the obtained properties as essential for the functionality of this protein family, but the final binding step may add further relevant details to the process-explanation.

The water molecules in the first hydration layer are bound to the surface of the solute *via* non-bonded interactions (van der Waals and Coulomb). The enthalpic interactions describe how strong a water molecule is bound to the protein surface. Therefore, if the protein-water interactions are strong, water molecules cannot leave their positions. One can imagine such a water molecule as being attached to the protein through a stiff, rigid spring. In contrast, when a molecule is only weakly bound, it is very likely able to move without a large enthalpic penalty (*i.e.* without a large, unfavorable change in free energy). We can picture this as a water molecule attached to the protein surface with a flexible spring.

**Figure 5:**
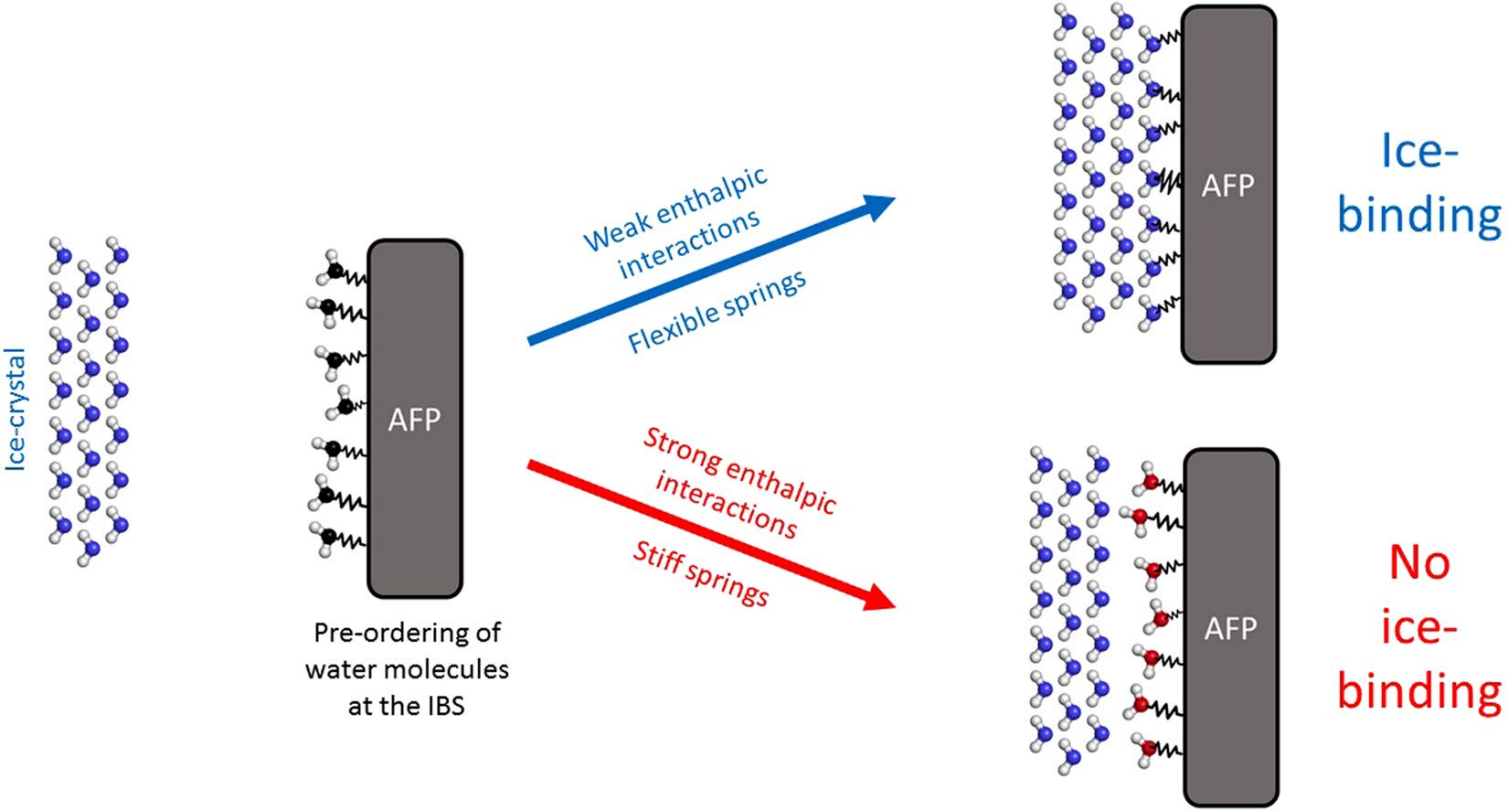
Schematic representation of the ice-binding process highlighting the influence of enthalpic interactions between the protein and the first hydration shell. Pre-ordering of the water molecules in an ice-like structure around the IBS (left); if the protein water interactions are too strong, the water molecules cannot rearrange and an energetically unfavorable mismatch between the ice and the protein occurs (bottom right). If the enthalpic interactions are weak, the springs are flexible and the water molecules are able to rearrange and fit to the ice lattice (top right).

This model can now be combined with the already established hypothesis of ice-like water molecules forming the first hydration shell, adding a more detailed explanation of the recognition and adsorption step. Water molecules in the first hydration layer form ice-like structures around the IBS. This ordering of water is not as perfect as in ice (schematically depicted on the left side of Fig. 5). Roughly, the correct distances have to be realized, as otherwise the initial ice-recognition is not possible. Therefore, a minimum amount of ice-like order is a prerequisite for the hydration layer of an IBS.

We propose that this ice-like pre-ordering of the first hydration shell of the protein, discussed extensively in the literature^29–31^ and not investigated within this study, is only the first step and subsequent reorganization of the water molecules is necessary to match the ice surface. The energy penalty for the rearrangement *E* can also be described in an approximative manner with the proposed spring model, it depends on the spring constant *k* and on the displacement from the equilibrium position ∆*x*.

1 $$E=-k{\rm{\Delta }}{x}^{2}$$

When the interactions between the water molecules and the protein are very strong, the water molecules are very tightly bound and the corresponding spring constant is large. In this case, the water molecules are not able to rearrange to a nearly perfect ice - shape, or only with a high enthalpic penalty. Thereupon, the binding between water molecules of the first hydration layer and the ice crystal is not favorable (bottom right of Fig. 5) as the energy to overcome the mismatch between the hydration layer and the ice surface is larger than the free energy gain upon binding. If enthalpic interactions are weaker and the springs are flexible, the spring constant is small, and rearrangement can occur (top right of Fig. 5). Hence, the water molecules can rearrange to an ideal ice - shape and bind to the growing ice crystal. As the rearrangement of loosely bound waters comes along with only a minor change of their enthalpy, the entropic gain upon binding and the free energy gain of the additional ice formation can overcompensate this enthalpy penalty.

Also the lattice match between the hydration shell and the ice surface plays an important role for the rearrangement energy: If the lattice mismatch between the ice lattice and the protein hydration is small, the expansion of the springs from their equilibrium distances Δ*x* is small and the enthalpic penalty is low.

Therefore, if the lattice match is good or even perfect, a higher interaction energy can be tolerated or can even be beneficial for ice-binding. As almost anywhere in nature, if the lattice match is not perfect, high interaction energies might hinder the process of ice-binding.

The simplistic view of the ice recognition problem, given in Fig. 5, agrees with previously examined properties of IBSs . The hitherto generated knowledge of a lattice match between the AFP and the ice surface being favorable for ice-binding is still valid for the mechanism presented here, because the energy penalty for the rearrangement also depends on the distance Δ*x* the atoms have to travel from their equilibrium position, and therefore, on the pre-ordering of an ice-like surface. Our thermodynamic picture adds a further requirement for ice-binding surfaces and can also explain why AFP ice-binding surfaces are rather hydrophobic and uncharged. Although the pre-ordering could be established with charged AAs, the enthalpic interactions would be too strong to allow for reorientation of the surface water molecules.

Our proposed mechanism also explains why at the NIBSs of AFPs very often charged residues are found. Instead of just increasing the hydrophilicity of the molecule, they also prevent further ice growing by the mechanism proposed and, therefore, prevent the protein from being completely coated by ice. Furthermore, our study explains why not the most hydrophobic residues are found in IBSs. These residues would have low solute water enthalpic interactions, favorable for the ice-binding, but they do not sufficiently pre-order water in their hydration shell. Therefore, we propose that the balance between entropy and enthalpy has to be within a precise range for an IBS. Very hydrophilic surfaces (e.g. charged amino acids) induce strong ordering in their surroundings, which is favorable for ice-binding, but also impose too strong enthalpic interactions hindering the process of ice-binding. Too hydrophobic surfaces on the other hand, show low enthalpic interactions with water but are not able to order the hydration layer ice-like^56^. Therefore, AAs, such as THR, VAL, ALA, and also GLY, which fulfill these requirements (entropy and enthalpy) are often found in AFPs.

A similar observation for a related chemical substance was reported by Michaelides and coworkers: They found that the hydrophobicity of a surface has to be in the correct range to show ice-nucleation activity^63,64^. Whereas ice-nucleation, at first glance, does not seem to be the best example to compare the AFPs’ behavior to, it is very likely that the mechanisms of these processes are very similar. Dolev *et al*. and Koop *et al*. already suggested that AFPs are very similar to ice-nucleation proteins (INPs), only differing in terms of protein size, or the size of the active site, respectively^19,65,66^. Small ice-binding proteins are good AFPs and show ice-nucleation activity only at very high concentrations^67^, whereas large INPs show nucleation activity at relatively high temperatures (−5 °C) but can also act partially as AFPs^68^. Additionally, Qiu *et al*. reported that the ice nucleation ability for alcohol monolayers is much better than for monolayers of acids of the same chain length, which agrees with our proposed mechanism. The interaction energies of acid functions are much higher than for alcohol groups and also the lattice mismatch is larger^56^. Interestingly, the group found that the ice nucleation ability increases with the interaction energy between the alcohol groups and the water molecules^62^. This might have multiple origins: Firstly, the optimal conditions, e.g., enthalpic interactions between water and solute, may be slightly different for AFPs, INPs and other ice nucleating agents, since AFPs could be more apolar than ice nucleating substances. Secondly, Qiu *et al*. only observed the increased nucleation ability at a perfectly matching surface; the effect may be different when the nucleus structures do not have a perfect ice-shape, such as the AFP surfaces investigated in this study. At these AFP surfaces, waters are only ice-like, their orientations differ from that of perfect ice and they have to rearrange to fit to the ice lattice. In nature, almost no materials are perfectly ice-shaped. For a perfect ice-shaped substrate these rearrangements are not required and mobility of the water molecules would hinder the ice nucleation ability of the material. Thirdly, we used an all atom force field in this study, which in general should describe the investigated structures in more detail.

As discussed, the mechanism for ice-nucleation is very likely to be similar to the one of AFPs. Therefore, Fig. 5 can be interpreted also for INPs. The pre-ordering in the hydration shell around the active species has to be ice-like, but not necessarily perfectly ice - shaped (Fig. 5 left), even an intermediate layer is possible, as shown by Pedevilla *et al*. for K-feldspar^69^. Due to thermal fluctuations, water can rearrange to form an ice-nucleus. Similar to AFPs, when the enthalpic interactions are too strong, these rearrangements are limited, whereupon no ice-nuclei can be formed (Fig. 5 right). In contrast, weak enthalpic interactions allow the formation of suitable ice-nuclei. This observation agrees with the studies of Lupi and Molinero, who showed that the ordering of the hydrating water is important^70^. It also agrees with studies by Li *et al*. finding the ice-nucleating ability of hydrophobic surfaces to be higher than of hydrophilic ones^71^. To summarize, low enthalpic interactions in combination with an entropy/enthalpy ratio as high as possible might be beneficial for the ice-binding activity of IBPs.

## Conclusion

With the help of MD simulations and GIST we were able to analyze the thermodynamic properties of AFP hydration. The IBSs and NIBSs of known active AFPs, as well as active and inactive mutations of an experimentally well-investigated AFP were studied. While for the often discussed entropic contribution no clear difference between the IBSs and inactive protein surfaces was found, it was possible to reveal a trend for the enthalpy gain, which is significantly lower for the active protein sites. Lower enthalpic interactions have their origin in weaker solute water interactions. Furthermore, we showed that a balance between entropy and enthalpy is key: only a suitable ratio is observed in IBPs. For all investigated proteins, IBSs show a higher entropy/enthalpy ratio than NIBSs or inactive proteins. Hence, active sites of AFPs introduces higher order in their hydration shell at a given solute water enthalpy compared to inactive sites.

Contributing an additional piece of enthalpic interactions to the already proposed mechanism of the ice-like water and entropy gain hypothesis, we were able to fine-tune the hitherto given explanation of the mechanism. Most notably, we added a further step to the proposed path, explaining why amino acids with strong solute-water interactions, like charged residues, are not able to bind to ice surfaces. This is in concordance with the studies of Qui *et al*., who found that the ability of acid monolayers to nucleate ice is weaker than for monolayers of alcohols^62^, and also of Zhang and Chen, who found that the icephobicity of smooth graphene is lower than for functionalized, more hydrophilic, graphene surfaces. Hence, icephobic surfaces might actually be hydrophilic^72^. This implies that the typically used hydrophobic lacquers (*e.g*. in aviation industry) support ice-binding, whereas different coating might help to prevent freezing at critical surfaces.

The results presented can also be applied to the mechanisms of other ice-binding proteins like ice-nucleation proteins^73^, ice-adhesion proteins^74^, and other ice-nucleating agents^75^. Our investigations thus help to understand the mechanism of ice-binding, an indispensable prerequisite for the design of antifreezing agents (*e.g*. lacquers for planes) and ice-nucleating materials (*e.g*. additives for snow cannons) but also for understanding processes during cloud formation.

## Methods

### Simulation Details

AFPs from the winter flounder (wfAFP), spruce budworm (sbwAFP) and mealworm (mwAFP) were simulated using the AMBER software package. X-ray single-crystal structures (1WFA^76^, 1L0S^77^, 1EZG^61^) were used as starting points. In the case of sbwAFP and mwAFP, two residues were not resolved in the crystal structures. Instead of these two AAs, capping groups (N-terminal acetyl (ACE) for sbwAFP and C-terminal N-methyl (NME) for mwAFP) were introduced. All mutations introduced to obtain the crystal structures were back-mutated to the naturally occurring form. wfAFP was, in addition to its naturally occurring form, also simulated in three mutated forms. For the mutants, the crystal structure 1WFA was used, too. Four THR residues (residue-id = 2, 13, 24, 35) were transformed to VAL, ALA, and SER accordingly. All proteins were solvated in an octahedral box using the AmberTools package with a minimum distance between the box edge and the protein of 12 Å^78^. The protein force field ff14SB^79^ was used in combination with the TIP4P-2005^80^ water model. TIP4P-2005 was chosen as it allows to reproduce properties of water molecules in the liquid as well as the solid state^81^. Furthermore, TIP4P-2005 shows a reasonable freezing point of 252 K, close to the experimentally measured value of 273 K. After solvation, the proteins were equilibrated according to a protocol developed by our group^82^. The protocol includes an intensive pre-equilibration of water, before the solute atoms can move. This prevents the artificial closing of protein pockets where no crystal water molecules are resolved. After equilibration, the proteins were simulated for 200 ns without restraints. A time step of 2 fs was used and coordinates were saved every 10 ps. To keep the pressure at 1 bar, an isotropic implementation of the Berendsen barostat was used^83^. To obtain more diverse structures, the temperature for the unrestrained simulations was set to 300 K for the AFPs from spruce budworm and mealworm *via* a Langevin thermostat^84^. For the winter flounder AFP, the temperature had to be set near the freezing point of the TIP4P-2005 water model (260 K), as otherwise the *α*-helical structure would have been degenerated.

Five representative conformations for each investigated AFP were obtained using a hierarchical agglomerative (bottom-up) clustering approach on these unrestrained MD simulations^85^. Five clusters obtained thereby were chosen, for the investigated proteins are rigid (especially sbwAFP and mwAFP), making a number of five adequate. The clusters were solvated and equilibrated, again according to the same protocol, but with coordinate restraints for the entire protein as required by GIST^86^. Every cluster was simulated for 100 ns; coordinates were saved every 100 ps for the GIST analysis. For the cluster simulations, a temperature of 260 K, close to the TIP4P-2005 freezing point, was chosen, as this is the temperature where the protein is active.

#### Grid Inhomogeneous Solvation Theory

The concept of GIST is briefly outlined in this section. For a more detailed discussion of the theoretical background we refer to respective publications of Gilson and co-workers^86,87^.

GIST aims at calculating the free energy of solvation for a fixed solute ΔG_Solv_(**q**). As restricting the solute to just one conformation is a rough simplification, multiple GIST calculations can be used to estimate the free energy of solvation of a flexible solute ΔG_Solv_ by summation (eq. 2).

2 $${\rm{\Delta }}{{\rm{G}}}_{{\rm{S}}{\rm{o}}{\rm{l}}{\rm{v}}}\approx \sum _{{\rm{q}}}{\rm{\Delta }}{{\rm{G}}}_{{\rm{S}}{\rm{o}}{\rm{l}}{\rm{v}}}({\bf{q}}){\rm{p}}({\bf{q}})$$

Here, p(**q**) corresponds to the probability to find the solute in conformation **q**. In this report, multiple possible conformations of the solute were accounted for by running unrestrained simulations of the proteins. Afterwards, the trajectory was subsequently clustered in five representative conformations. The five clusters were then simulated individually with positional restraints as required by GIST.

GIST allows to split the free energy in multiple enthalpic and entropic contributions as shown in eq. 3.

3 $${\rm{\Delta }}{{\rm{G}}}_{{\rm{S}}{\rm{o}}{\rm{l}}{\rm{v}}}\approx {\rm{\Delta }}{{\rm{E}}}_{{\rm{S}}{\rm{W}}}+\Delta {{\rm{E}}}_{{\rm{W}}{\rm{W}}}-{\rm{T}}{\rm{\Delta }}{{\rm{S}}}_{{\rm{t}}{\rm{r}}{\rm{a}}{\rm{n}}{\rm{s}}}-{\rm{T}}{\rm{\Delta }}{{\rm{S}}}_{{\rm{o}}{\rm{r}}{\rm{i}}{\rm{e}}{\rm{n}}{\rm{t}}}$$

The free energy is divided into two enthalpic and two entropic terms. The enthalpic terms cover the interaction energy between the solute and the water molecules ΔE_SW_, and the interaction between the water molecules ΔE_WW_. The solvation entropy consists of a translational ΔS_trans_ and an orientational part ΔS_orient_, covering the entropy of the water molecules. All terms depicted in eq. 3 are state functions and therefore need a reference state; in our case a simulation of pure water without any solute. The reference value for the water-water interaction is 12.01 kcal/mol, whereas the reference values for the remaining terms ΔE_SW_, ΔS_trans_, ΔS_orient_ are zero.

In addition, GIST uses a grid-based approach, which allows to obtain a spatial resolution of the solvation properties around a solute. This permits the investigation of the thermodynamic contributions to different protein sites.

The GIST output can be further processed to determine the structural properties of water on different protein surfaces. For further analysis, structural motifs of the different proteins were used. We made a distinction between the protein sites binding to ice and sites exposed to liquid solvent after binding, which are called “non ice-binding sites” in the following.

**Figure 6:**
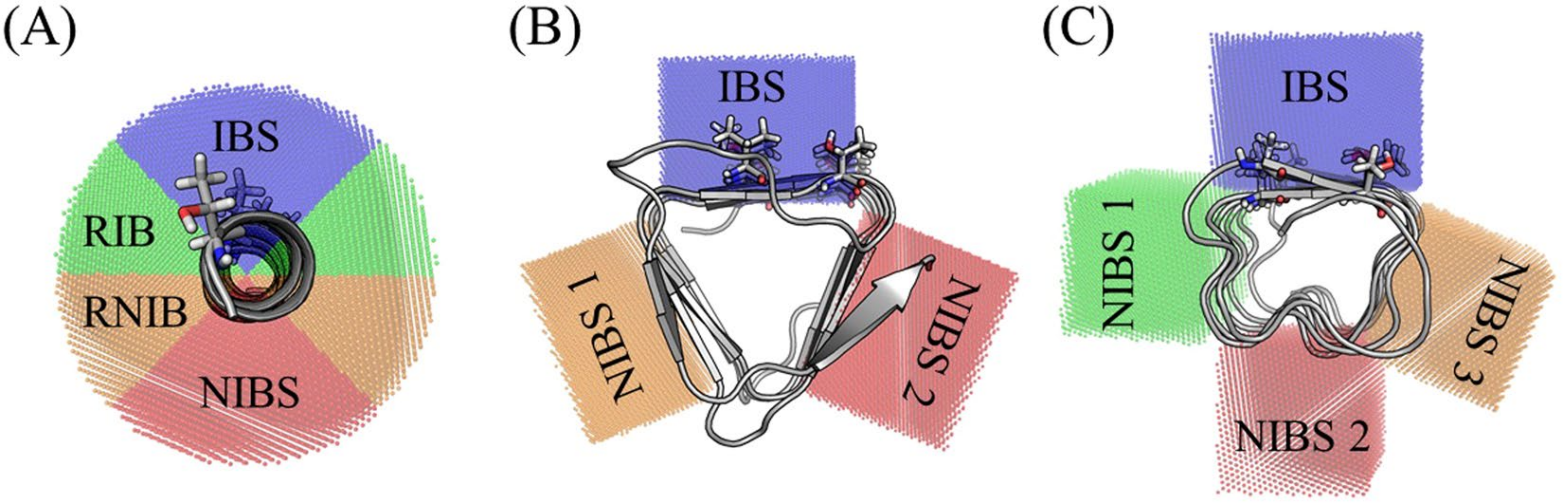
Three AFPs in cartoon representation: (**A**) wfAFP (**B**) sbwAFP (**C**) mwAFP. The residues in the active sites are shown in stick representation. The colored sections show the regions where water properties were analyzed, “active” ice-binding regions are given in blue. Remaining sites are named as rather ice-binding (RIB), rather non ice-binding (RNIB), and non ice-binding site (NIBS), respectively.

wfAFP consists of one *α*-helix only, making it a “quasi-cylindrical” system. Therefore, the surrounding water was divided in four regions (Fig. 6A). Four regions were chosen as a compromise; a smaller number does not capture the different sites of the AFP, whereas a larger number decreases the number of water molecules in the region, thereby introducing noise. As experimental studies had already shown that the four THRs play an active role in the binding process, it is established that the protein binds with the THR containing site (blue region in Fig. 6A) to the ice surface^61^. On the opposite side of the protein, we defined a region, which is definitely solvent exposed after binding: the non ice-binding site. Furthermore, two additional intermediate regions were defined: the first one, closer to the ice-binding region, is called “rather ice-binding site”, the second one closer to the non ice-binding region is called “rather non ice-binding site” in continuation.

For the spruce budworm AFP, it is possible to define three planes, as the protein has a triangular shape (see Fig. 6B)^88^. Through modification studies, the IBS (blue area in Fig. 6B) is also known for this protein. Thus, two NIBSs (red and orange regions) can be defined.

For mwAFP, we used a similar approach to divide the surrounding water. Instead of three distinct regions, four regions were defined for this protein due to its rectangular shape (Fig. 6C). Again, the experimentally identified IBS^89^ is colored in blue, whereas the three NIBSs are given in red, orange, and green, respectively.

The water properties in the colored regions were analyzed for all three proteins. For the blue circle segment of the wfAFP, the radius was set to 10 Å (from the center of the *α*-helix). For all other regions, the radius was set so that the same number of water molecules was analyzed as for the IBS region (approx. 28 water molecules, radii between 9 and 11 Å). Similar approaches were used for sbwAFP and mwAFP, however, the minimum distance to the plane, defined by the backbone atoms of the AAs building the surface, was used instead of the minimum distance to the *α*-helix. Again, the number of water molecules within a 10 Å layer of the IBS was used as reference value for all other regions. For sbwAFP and mwAFP different combinations of length and width for the integrated regions were tested, whereby the qualitative results remained the same. The reported values correspond to a base area of 14 × 14 Å^2^ for sbwAFP and 20 × 10 Å^2^ for mwAFP.

An approach based on highly occupied water positions was used to identify water sites of special interest close to the protein surface. Subsequently, the density of one water molecule was subtracted from the highest occupied grid point and its surrounding points and a water molecule was placed at this position. This results in a visualization of the most probable water positions and their corresponding thermodynamic values. A more detailed explanation of how these water positions were derived is given in our respective previous report^90^.

## Associated Content

### Data Availability

The datasets generated during and/or analyzed during the current study are available from the corresponding author on reasonable request.
